# *pdh* modulate virulence through reducing stress tolerance and biofilm formation of *Streptococcus suis* serotype 2

**DOI:** 10.1080/21505594.2019.1631661

**Published:** 2019-06-24

**Authors:** Yang Wang, Yuxin Wang, Baobao Liu, Shaohui Wang, Jinpeng Li, Shenglong Gong, Liyun Sun, Li Yi

**Affiliations:** a College of Animal Science and Technology, Henan University of Science and Technology, Luoyang, China; b Key Laboratory of Molecular Pathogen and Immunology of Animal of Luoyang, Luoyang, China; c Shanghai Veterinary Research Institute, Chinese Academy of Agricultural Sciences, Shanghai, China; d College of Life Science, Luoyang Normal University, Luoyang, China

**Keywords:** Streptococcus suis, pyruvate dehydrogenase, virulence, adhesion, biofilm formation, stress

## Abstract

*Streptococcus suis* serotype 2 (*S. suis* 2) is a zoonotic pathogen. It causes meningitis, arthritis, pneumonia and sepsis in pigs, leading to extremely high mortality, which seriously affects public health and the development of the pig industry. Pyruvate dehydrogenase (PDH) is an important sugar metabolism enzyme that is widely present in microorganisms, mammals and higher plants. It catalyzes the irreversible oxidative decarboxylation of pyruvate to acetyl-CoA and reduces NAD+ to NADH. In this study, we found that the virulence of the *S. suis* ZY05719 sequence type 7 *pdh* deletion strain (Δ*pdh*) was significantly lower than the wild-type strain (WT) in the mouse infection model. The distribution of viable bacteria in the blood and organs of mice infected with the Δ*pdh* was significantly lower than those infected with WT. Bacterial survival rates were reduced in response to temperature stress, salt stress and oxidative stress. Additionally, compared to WT, the ability to adhere to and invade PK15 cells, biofilm formation and stress resistance of Δ*pdh* were significantly reduced. Moreover, real-time PCR results showed that *pdh* deletion reduced the expression of multiple adhesion-related genes. However, there was no significant difference in the correlation biological analysis between the complemented strain (CΔ*pdh*) and WT. Moreover, the survival rate of Δ*pdh* in RAW264.7 macrophages was significantly lower than that of the WT strain. This study shows that PDH is involved in the pathogenesis of *S. suis* 2 and reduction in virulence of Δ*pdh* may be related to the decreased ability to resist stress of the strain.

## Introduction

*Streptococcus suis* (*S. suis*) is a serious zoonotic pathogen that causes meningitis, arthritis, pneumonia and sepsis in pigs, leading to extremely high mortality. *S. suis* serotype 2 is most often associated with disease in Asia [1–3]. Moreover, people can be infected, and a high proportion of streptococcal toxic shock syndrome (STSS) occurs in patients [4,5]. *S. suis* infection in the host usually causes symptoms of fever and inflammation. Although these symptoms can cause damage to the body, they are also an effective method for the host to kill the invading bacteria. This process is mainly achieved by changes in temperature, osmotic pressure, oxidizing power and pH. Therefore, the pathogenicity of *S. suis* 2 is strongly related to its ability to resist stress. *S. suis* 2 infection has become one of the three most important diseases of pig farms. It has caused serious economic losses and even poses a serious threat to human life. However, the pathogenesis of *S. suis* 2 is still not fully understood.

Pyruvate dehydrogenase (PDH) is a multi-enzyme complex widely found in microorganisms, mammals, and higher plants. PDH comprises a group of rate-limiting enzymes that catalyze the irreversible oxidative decarboxylation of pyruvate to acetyl-CoA, while reducing NAD+ to NADH and linking the anaerobic glycolysis of sugars to the tricarboxylic acid cycle, providing energy through oxidative phosphorylation [6]. Studies have also shown that PDH is also involved in multiple functional regulation. In a previous comparative proteomic study, we found that the expression of PDH in *S. suis* in the biofilm state was about 2.5-fold higher than in the planktonic state [7]. The result is consistent to other reports [8,9]. Moreover, PDH is thought to play an important role in bacterial adhesion to the extracellular matrix and is an important component of the bacterial cytoskeleton [10,11]. To learn about the involvement of *S. suis pdh* in pathogenesis, we constructed a *S. suis pdh* gene deletion mutant (Δ*pdh*) and complemented strain (CΔ*pdh*). We analyzed and compared adhesion, invasion, biofilm formation and stress response of WT, Δ*pdh* and CΔ*pdh* strains.

Collectively, the results showed that the loss of *pdh* leads to a decrease in the adhesion and invasion of *S. suis*, a decrease in biofilm formation and easy to be cleared by macrophages. We also showed that Δ*pdh* had a decreased ability to resist heat stress, salt stress and oxidative stress, but resistance to acid stress was not significantly to WT. The present study for the first time showed that PDH has an important influence on the pathogenicity of *S. suis* 2.

## Results

### Confirmation of the pdh knockout mutant strain

To investigate the PDH function in *S. suis*, we amplified the two DNA fragments flanking the *pdh* gene and successfully constructed the homologous suicide recombinant plasmid pSET4s-*pdh*. The plasmid was then transformed into *S. suis* ZY05719 and positive transformants were screened by the presence of spectinomycin and temperature changes. The construction of the Δ*pdh* strain was confirmed by PCR analysis. In order to more accurately verify the function of PDH in *S. suis*, we also successfully constructed CΔ*pdh* strain using pSET2s as a shuttle plasmid (data not shown).

### Growth phenotype of δpdh

**Figure 1.**
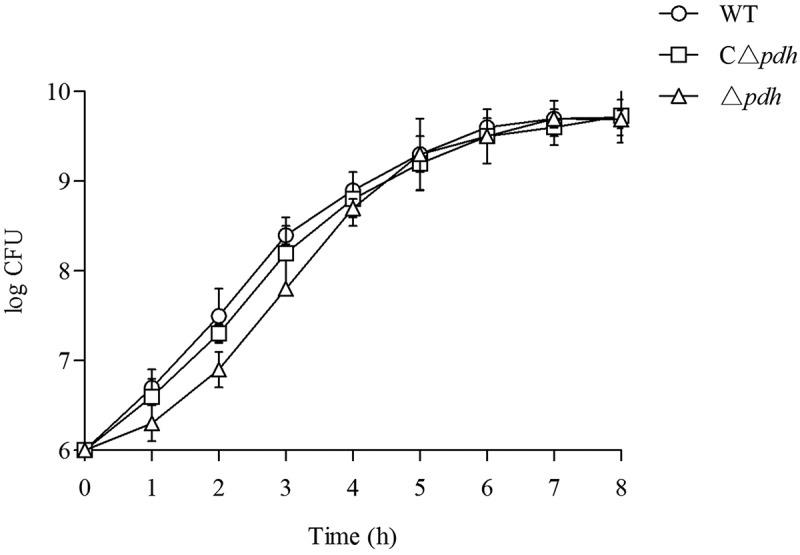
Growth of the WT, Δ*pdh* and CΔ*pdh S. suis* strains in THB medium. (a) Growth was assessed by determination of OD_600nm_ values at the time points indicated. (b) Growth was assessed by determination of viable counts at the time points indicated. Each time point represents three independent tests.

As shown in Figure 1, when the viable counts of the WT, Δ*pdh* and CΔ*pdh* strains were determined during the above growth assays, it was found that there was no significant difference in CFU numbers among the three strains.

### LD50 and virulence of WT and δpdh in mice

As shown in Table 1, the LD_50_ of the WT was 2.245 × 10^5^ CFU. Meanwhile, the LD_50_ of Δ*pdh* was 3.874 × 10^6^ CFU and the LD_50_ of CΔ*pdh* was 1.062 × 10^5^ CFU. These results indicate that Δ*pdh* virulence was weakened (*P* < 0.01), while the virulence of CΔ*pdh* was restored, with values 36.48-fold higher than Δ*pdh*.

**Table 1.** Calculations of LD_50_ on *S. suis* and its derivatives for mice.

Group	CFU Per mouse				Mortality				LD_50_/(CFU)
WT	2.587 × 10^7^	3.014 × 10^6^	2.601 × 10^5^	3.532 × 10^4^	9/10	8/10	5/10	3/10	2.245 × 10^5^
Δ*pdh*	1.547 × 10^8^	2.12 × 10^7^	2.14 × 10^6^	2.734 × 10^5^	9/10	6/10	5/10	2/10	3.874 × 10^6^
CΔ*pdh*	2.694 × 10^7^	2.876 × 10^6^	2.735 × 10^5^	3.621 × 10^4^	10/10	8/10	6/10	4/10	1.062 × 10^5^

### Viable bacteria in blood and organs

**Figure 2.**
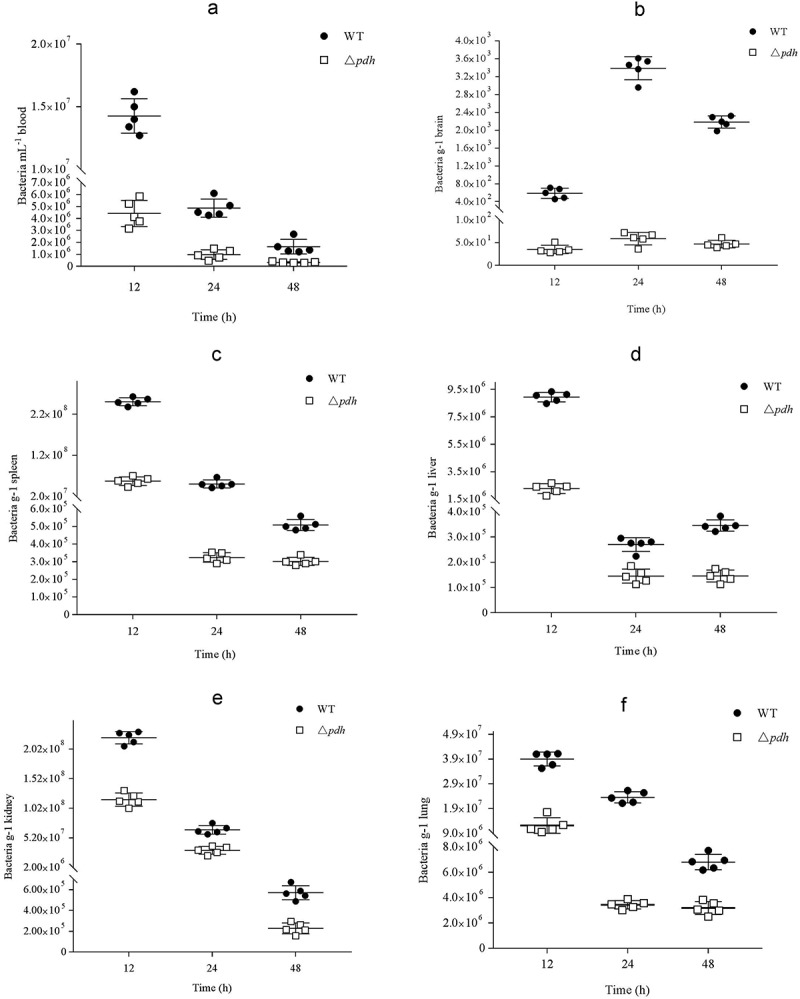
Bacterial counts in different organs at different post infection time. (a) blood; (b) brain; (c) spleen; (d) liver; (e)kidney; (f) lung.

After the mouse blood, brain, spleen, liver, kidney and lung were taken, the distribution of bacteria in various organs in mice was analyzed. As shown in Figure 2, there was a significant difference in the bacterial content of the WT and Δ*pdh* strains. The bacterial counts for Δ*pdh* were significantly lower than the WT (*P* < 0.01).

### Roles of PDH in stress tolerance of S. suis

**Figure 3.**
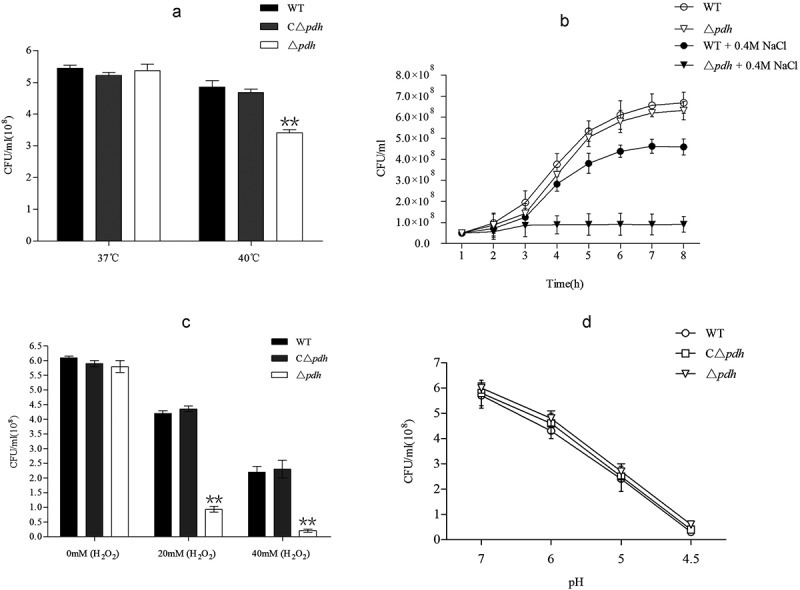
In vitro stress assays of WT, Δ*pdh* and CΔ*pdh* strains.(a) heat stress assay; (b) osmotic stress assay; (c) oxidative stress assay; (d) acid stress assay. The assays were performed in duplicate and repeated three times. Significant differences are indicated.

The magnitude of the anti-stress ability of the bacteria directly determines their survival. To further assess the role of PDH in stress tolerance, we exposed WT, Δ*pdh* and CΔ*pdh* strains to various stress challenges. As shown in Figure 3, the results showed that the viable count of Δ*pdh* was lower than that of WT at 40°C for 12 h (*P* < 0.01). The results of salt stress test showed that the growth of Δ*pdh* strain decreased in 0.4M NaCl significantly than that of WT (*P* < 0.01). The WT and Δ*pdh* strains were treated with different concentrations of H_2_O_2_ and the results showed that the Δ*pdh* strain was more sensitive to H_2_O_2_ using 20mM and 40mM H_2_O_2_ (P < 0.01). The results of acid stress test showed that WT and Δ*pdh* viability decreased with pH value of the medium. However, there was no significant difference between the three strains (*P* > 0.05). It is worth noting that there was no significant difference in stress tolerance between WT and CΔ*pdh* in various stress assays (*P* > 0.05).

### PDH deficiency inﬂuences biofilm formation

**Figure 4.**
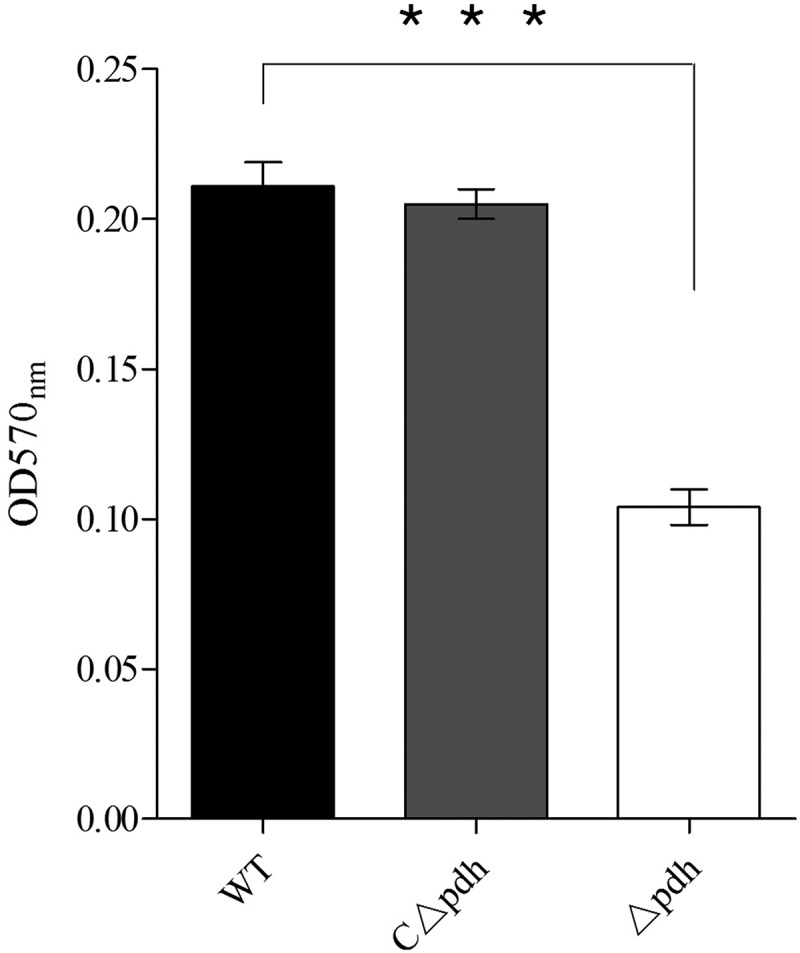
Quantitative microtiter plate assay for biofilm production of WT, Δ*pdh* and CΔ*pdh* strains. The assay was performed in duplicate and repeated three times. Significant differences are indicated.

Biofilms are an important protective mechanism for the survival of microorganisms and their ability to form biofilms can affect persistence in the host and antibiotic resistance. In the present study, we found that the biofilm forming ability of the Δ*pdh* strain was significantly weakened compared to the WT and the CΔ*pdh* strains (*P* < 0.001). It is suggested that *pdh* is involved in the formation of *S. suis* biofilms (Figure 4).

### PDH is involved in adherence and invasion in vitro

**Figure 5.**
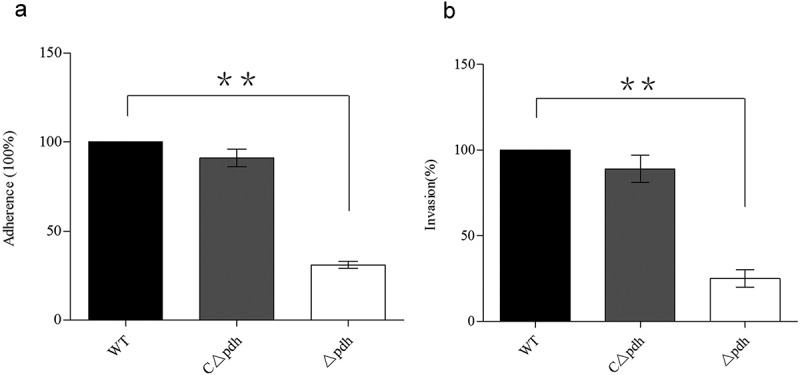
Adhesion and invasive ability of WT, Δ*pdh* and CΔ*pdh* strains. The adhesion and invasive ability of WT was used as a reference to determine the changes in adhesion and invasive ability of Δ*pdh* and CΔ*pdh*. The columns represent the means and standard deviations of three or more experiments.

To evaluate the effect of PDH deficiency on bacterial adhesion and invasion, we examined the adhesion and invasion ability of the Δ*pdh* strain in PK-15 cells. As shown in Figure 5, the adhesion and invasion ability of the Δ*pdh* strain to PK-15 cells was significantly reduced compared to the WT (*P* < 0.01). There was no significant difference in adhesion and invasion of PK-15 cells between WT and CΔ*pdh* strains (*P* > 0.05).

### Adhesion gene transcription analysis

**Figure 6.**
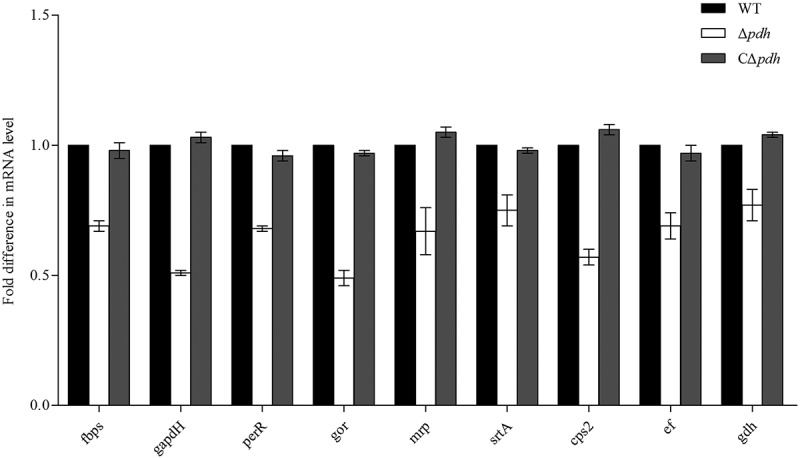
Expression of *S. suis* adhesion genes. Total RNA of WT, Δ*pdh* and CΔ*pdh* strains was extracted for qPCR. The 16S RNA gene was used as a reference. The figure shows that the gene expression level in the WT strain is 100%, and the gene expression in the Δ*pdh* and CΔ*pdh* strains were the relative to expression in the WT strain genes. Data from three independent assays are expressed as mean ± SD.

The results of the Δ*pdh* adhesion test indicated that PDH was involved in the adhesion of *S. suis*. To further understand the relationship between PDH and adhesion-related factors, we analyzed the expression profiles of several adhesion related genes by qPCR. Compared to the WT, the results showed that the expression levels of the adhesion-related genes *ccpA, fbps, gapdH, perR, gor, mrp, srtA, cps2, ef* and *gdh* were significantly decreased in theΔ*pdh* strain by 0.69, 0.51, 0.68, 0.49, 0.67, 0.75, 0.57, 0.69 and 0.77 (*P* < 0.05). However, there was no significant difference between the WT and the CΔ*pdh* strains (*P* > 0.05) (Figure 6).

### PDH is involved in bacterial survival in phagocytic cells

**Figure 7.**
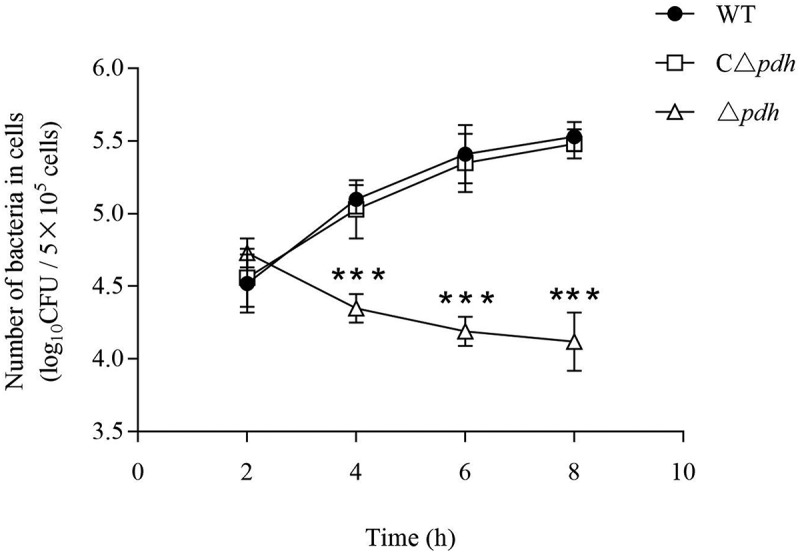
Intracellular growth of the *S. suis* 2 WT, Δ*pdh* and CΔ*pdh* strains in RAW264.7 macrophages. The assay was performed in duplicate and repeated three times. Significant differences are indicated.

To evaluate the effect of PDH in the interaction between bacteria and phagocytic cells, we examined the phagocytosis assay between Δ*pdh* strain and RAW264.7 cells. the results showed that the survival of Δ*pdh* strains in RAW264.7 cells was significantly decreased at 4 h, 6 h and 8 h compared with WT and CΔ*pdh* strains, and the difference was extremely significant (*P* < 0.001), while there was no significant difference between WT and CΔ*pdh* strains (*P* > 0.05) (Figure 7).

## Discussion

PDH is an enzyme present in the mitochondria of microorganisms, and mammals and is responsible for the oxidative decarboxylation of pyruvate to form acetyl CoA (Acetyl-CoA), while reducing NAD+ to NADH [12,13]. Many studies have found that enzymes are involved in the regulation of bacterial virulence and biofilm formation in addition to their inherent functions [2,14]. At present, research on PDH has mainly focused on carbohydrate metabolic pathways [15,16]. However, little research has been done on the role of PDH in microbial pathogenesis. We have constructed a *S. suis* Δ*pdh* deletion mutant and complemented strain. This study further clarified the important role of PDH in the pathogenesis of *S. suis*.

The pathogenic process of *S. suis* infection of the host is complicated and is accomplished by multi-step and multi-component synergy. Among these, colonization of host cells is considered to be a critical step. In order to better study the role of PDH in *S. suis*, we performed LD_50_ detection and organ infection experiments in mice. The virulence of the Δ*pdh* strain was decreased by 17.26-fold compared to WT strain. The results demonstrate that inactivation of *pdh* reduces the virulence of *S. suis* in mice. The adhesion and invasion of *S. suis* in the blood and tissues of the host is a key factor in the pathogenesis of bacterial infections [17,18]. The distribution of viable counts in different tissues was significantly lower than that of the WT strain. The above experimental results show that PDH has an effect on the virulence of *S. suis*.

After the bacteria infect the host, the host often resists the bacteria through changes in the internal environment [19]. According to the environmental stresses that *S. suis* may face during the infection, four in vitro simulated stress conditions were tested with reference to previous methods: high temperature (37°C and 40°C), salt stress (0.4M NaCl), oxidative stress (0 mmol/L, 20 mmol/L, 40 mmol/L H_2_O_2_) and acid stress (pH 7.0, 6.0, 5.0, 4.5). The results showed that compared with WT, the growth of Δ*pdh* at high temperature, high osmotic pressure and high oxidation was significantly reduced, while there was no significant difference between CΔ*pdh* and WT. At the same time, under acid stress, the growth of the three strains was reduced and there was no significant difference. This indicates that *pdh* was up-regulated under temperature stress, salt stress and oxidative stress. The results of this study suggest that an increase in LD_50_ of Δ*pdh* may be related to the decreased ability to resist stress of the strain.

Biofilm formation is an important process by which bacteria establish infections [20,21]. At the same time, biofilms also protect bacteria against harsh environments [22]. In this study, the results showed that *S. suis* biofilm formation was significantly decreased after PDH deletion compared with WT. Biofilm formation is strongly related to the ability of bacteria to adhere [23]. Studies have found that *Lactobacillus plantarum* pyruvate dehydrogenase (PDHB) is not only involved in biofilm formation but is also a collagen-binding adhesin. Deletion of PDHB results in decreased binding ability of *L. plantarum* to collagen [24]. We selected porcine renal epithelial cells PK-15 to assess the role of PDH in bacterial adhesion and invasion. The results showed that the loss of *pdh* caused a significant decrease in the ability of *S. suis* to adhere and invade PK-15 cells. These results are similar to that of Marzia [24]. However, how does the loss of the *pdh* gene cause a decrease in the ability of *S. suis* to adhere and invade PK-15 cells? Studies have shown that bacteria have multiple genes involved in adhesion, such as *ccpA, fbps* and *gapdh* [25–27]. In the present study, we determined that the loss of PDH caused significant changes in the expression levels of 10 adhesion-related genes, including *ccpA, fbps, gapdH, perR, gor, mrp, srtA, cps2, ef* and *gdh*. The results indicate that PDH may be involved in the expression of adhesion-related factors, thereby reducing the ability of *S.suis* to adhere and invade PK-15 cells. It may be that PDH is involved in signaling as a result of adhesion–ligand binding by professional sensor systems [28]. The relevant mechanism requires further research. However, based on the results of our biofilm formation, cell adhesion, cell invasion and qPCR assays, it was confirmed that PDH plays an important role in the survival and colonization of *S. suis*.

The interaction between bacteria and macrophages is also an important part of the body’s defense against bacterial infections, usually responsible for phagocytosis and digestion of pathogens. Mouse macrophage RAW264.7 is a very important cell, and multiple studies have examined the interaction of this cell with bacteria [29–31]. In this study, compared with WT and CΔ*pdh* strains, the survival of Δ*pdh* strains in RAW264.7 cells decreased significantly at 4h, 6h and 8h, the difference was extremely significant (*P* < 0.001), while there was no significant difference between WT and CΔ*pdh* strains (*P* > 0.05). The results of intracellular survival assay indicate that *S. suis* is more easily cleared by macrophages after deletion of the *pdh* gene.

In summary, we have demonstrated for the first time that the inactivation of PDH, which is involved in the survival and virulence of *S. suis* 2 in animals, decreased the ability of adhesion, biofilm formation and anti-phagocytic. Furthermore, bacterial survival rates were reduced in response to temperature stress, salt stress and oxidative stress. The results of this study indicated that PDH deficiency reduces the pathogenicity of *S. suis* 2.

## Materials and methods

### Bacterial strains and culture conditions

*S. suis* 2 ZY05719 sequence type 7 WT strain was isolated from a sick pig during an epidemic outbreak in 2005 in Sichuan, China [32]. Under normal conditions, the *S. suis* was in Todd-Hewitt Broth (THB) (Difco Laboratories, Detroit, MI) or on Todd-Hewitt agar (THA) at 37°C. Chemicals were purchased from Sigma-Aldrich (China) unless otherwise stated. *Escherichia coli* (*E. coli*) strain DH5α was cultured in Luria-Bertani (LB) medium or on LB solid plates containing 2% agar. When appropriate, spectinomycin at a concentration of 100 mg/mL or ampicillin at a concentration of 50 mg/mL was added.

### Ethics statement

All animal experiments in this study were approved by the Experimental Animal Monitoring Committee of Henan University of Science and Technology and carried out accordingly.

### Construction of a pdh-deficient mutant (δpdh) and its complement strain (Cδpdh)

The *pdh* gene of *S. suis* was knocked out as previously described [33]. The strains and plasmids used in this study are listed in Table 2. And all primers used to construct mutant strains are listed in Table 3. Briefly, *pdh* was deleted in *S. suis* by allelic exchange using the thermal suicide vector pSET4s. First, two DNA fragments flanking the *pdh* gene were amplified from the genome of ZY05719 by PCR, and then the homologous recombination fragment containing no *pdh* gene was amplified by fusion PCR using the primer Tup/Sdown. After digestion with *Sal* I and *EcoR* I restriction enzymes, the homologous recombinant fragment was cloned into the vector pSET4s to generate the *pdh* knockout plasmid pST4s-*pdh*. To obtain the isogenic mutant Δ*pdh*, the recombinant plasmid pST4s-*pdh* was transformed into the prepared ZY05719 competent cells by electroporation as described earlier [33]. After two-step allelic exchange, spectinomycin-sensitive clones were selected. Then, the genomic DNA (gDNA) was analyzed using the lateral identification primers *pdh*-X/*pdh*-Y and the internal identification primer *pdh*-ORF-S/*pdh*-ORF-A, and then the PCR product was subjected to DNA sequencing to confirm the correct characteristics of the *pdh* mutant. *S. suis* WT strain was used as a negative control. The complement strain CΔ*pdh* was constructed on the Δ*pdh* background with pSET2 vector as previously described [33].

**Table 2.** Bacterial strains and plasmids used in this study.

Strains/plasmids	Relevant characteristics	Source of references
Strains		
*S. suis* ZY05719	One of the most toxic strains; isolated from dead pig	Collected in our laboratory
Δ*pdh*	ZY05719 has no PDH functional strain	This work
CΔ*pdh*	Complemented strain of ZY05719 Δ*pdh*	This work
*E. coli* DH5α	DH5a is a strain commonly used for plasmid cloning	Collected in our laboratory
pMD18-T	Cloning vector	TaKaRa
pSET4s	Thermosensitive suicide vector; spectinomycin resistant	Collected in our laboratory (Takamatsu et al., 2001b)
pSET2	*E. coli*–*S. suis* shuttle vector; spectinomycin resistant	Collected in our laboratory (Takamatsu et al., 2001a)

**Table 3.** Primers used in this study.

Primers	Sequence (5′-3′)	Source of references
Tup	CCGCGTCGACAATTCTTTATCCAATGGGAT (*Sal*I)	This work
Tdown	GGTGTGAGGACGTTAAAGAG (1032bp)	
Sup	CTCTTTAACGTCCTCACACCTTTCAAGAACATGTCCAAAT	This work
Sdown	ACCGGAATTCAAAAGGTCTGAGTTGACGAA (*EcoR*I）(958bp)	
*pdh*-X	CCAATGGGATAAGGGTA	This work
*pdh*-Y	TGTTTCATTGACGAGTAAA (2685 bp)	
*pdh*-ORF-S	CATGATGGCTGAGCTTGC	This work
*pdh*-ORF-A	TAAGCGTGTTCTTTCGGG (681 bp)	
*pdh*-F	CGCGAATTCATGCAACAAATCCGTGAT (*EcoR*I）	This work
*pdh*-R	CCCTCGAGGCTAGTCTACAAACACATC (*Xho*I) (924bp)	

### Growth phenotype of WT, δpdh and Cδpdh strains

Bacterial growth and phenotype analysis of strains were performed as previously described with small modifications [34]. Overnight cultures of the WT, Δ*pdh* or CΔ*pdh* strains with a final concentration of approximately 10^6^ CFU/mL were transferred to fresh THB medium and cultured at 37°C. Samples were estimated via dilution plating at 1 h, 2 h, 3 h, 4 h, 5 h, 6 h, 7 h and 8 h to measure the number of viable cells at each time point. Samples were subjected to vigorous vortexing before plating for viable counting to ensure that any long chains of organisms were broken up.

### Virulence assay

Taking the cultures of WT, Δ*pdh* and CΔ*pdh*, and the bacteria were washed with physiological saline and diluted to 10^4^ CFU, 10^5^ CFU, 10^6^ CFU, 10^7^ CFU or 10^8^ CFU, and 10 specific pathogen-free (SPF) female BALB/c mice (4 weeks old) were intraperitoneally injected for each group. Fourteen days after the challenge, the number of dead mice was recorded and the LD_50_ results were calculated.

### Determination of viable bacteria in organs

By detecting the degree of bacterial infection and the distribution of bacteria between different tissues after *S. suis* infection, tissue damage to the host and the main organs of infection can be determined. The in vivo bacterial survival assay was performed as described previously with slight modifications [35,36]. Briefly, WT or Δ*pdh* cells in logarithmic growth were resuspended in saline, diluted to 5 × 10^6^ CFU/mL, and 1 mL of the bacterial solution was intraperitoneally injected into each of 25 SPF female BALB/c mice (4 weeks old). Another 5 mice were injected with normal saline as a negative control. Blood samples (100 μL) were collected from the tail vein at 12 h, 24 h and 48 h postinfection. Five mice per group were euthanized at 12 h, 24 h and 48 h after infection. The 100 μL blood samples and the same weight of brain, spleen, liver, kidney and lung were homogenized, diluted and plated onto THB agar to determine the number of bacterial colonies. Colonies were counted and expressed in colony forming units (CFU)/mL.

### In vitro stress assays

The heat stress assay was performed as previously described with small modifications [37]. The WT, Δ*pdh* and CΔ*pdh* cells were transferred to 5 mL of THB liquid medium and cultured overnight. The cultures were diluted with fresh THB liquid medium in the ratio of 1:100 and cultured in a 37°C or 40°C incubator for 12 h. 100μL of the bacterial solution was serially diluted 10 times and 100 μL of the various dilutions were plated onto THB agar plates. Colonies were counted after incubation at 37°C for 24 h. Assays were performed in triplicate in independent trials.

To assess the adaptability of the *S. suis* to osmotic stress, bacterial growth kinetics were measured in THB containing 10% N-bromosuccinimide (NBS) and 0.4 mol/L NaCl as described previously with small modifications [36]. The cultures were diluted in fresh medium with or without NaCl liquid medium in the ratio of 1:100 and incubated for 8 h at 37°C. Bacterial growth was monitored by measuring viable counts at 1 h intervals. The assays were performed in triplicate and repeated three times.

The oxidative stress assay was performed in accordance with previously described methods [38]. The WT, Δ*pdh* and CΔ*pdh* strains were cultured to logarithmic growth phase in THB medium. H_2_O_2_ was added to the cultures (either 20 or 40 mM final concentration) and incubated at 37°C for 20 min. Each reaction was then diluted and each culture was plated. Bacteria incubated for 20 min in media without H_2_O_2_ were used as a reference. The results are expressed as survival rates. The assay was performed in triplicate and repeated three times.

The ability of different strains to resist acid killing was determined using a previously described method [39]. The WT, Δ*pdh* and C△*pdh* strains were harvested at mid exponential phase and centrifuged at 4000 × g for 10 min at 4°C, then washed once with glycine buffer (0.1 mol/L, pH 7.0). Cells were resuspended in THB liquid medium at different pH values (7.0, 6.0, 5.0, 4.0, 3.0) and incubated at 37°C for 45 min. 100 μL of the bacterial solution was serially diluted 10 times and 100 μL of the various dilutions were plated on THB plates. Colonies were counted after incubation at 37°C for 24 h. The differences in tolerance of the WT, Δ*pdh* and CΔ*pdh* to acid were compared. The experiments were performed in triplicate and repeated at least three times.

### Bioflm formation assay

Biofilm formation assays were performed according to previously described methods [40]. The cultures were diluted with fresh THB liquid medium in the ratio of 1:100. Experiments were divided into 3 groups and 200 μL of the diluted culture from each group was added to a 96-well microplate. After incubation at 37°C for 24 h, the cultures were removed and washed three times with sterile PBS to remove all planktonic cells, then fixed with 200 μL of methanol. After air drying, 200 μL of crystal violet was added for staining and washing. After complete drying, 200 μL of 95% alcohol was added to each well to dissolve the crystal violet and the 96-well plate was shaken gently for 10 min to completely dissolve the biofilm. Absorbance was recorded at 570 nm. The experiments were run in triplicate and repeated at least three times.

### Adherence and invasion assays

The disease caused by *S. suis* in the host begins with the colonization of epithelial cells and subsequent invasion of the bloodstream [41]. Porcine kidney epithelial (PK-15) cells are a subcultured cell line that is isolated and domesticated in pig kidneys. Studies have used PK-15 cells to detect the ability of *S. suis* adhesion and invasion [42–44]. To assess the ability of *S. suis* to adhere to PK-15 cells after deletion of *pdh*, we performed adhesion and invasion assays according to previously described methods [41]. PK-15 cells (1.6 × 10^5^ cells per well) were infected with log-phase WT, Δ*pdh* or CΔ*pdh* bacteria (OD_600nm_ 0.8) at a multiplicity of infection (MOI) (bacteria: cells) of 100:1, and placed in a 37°C 5% CO_2_ incubator for 2 h. To measure adhesion, 0.1% trypsin (200 μL) was added to each well for 5 min at room temperature and the cells were counted by plate counting after lysis. To determine the invasive ability of *S. suis*, 100 U/mL penicillin and 0.1 mg/mL streptomycin in 1 mL of sterile phosphate buffer were added per well. After 45 min at room temperature, wells were washed three times with PBS to remove all bacteria outside the cells. The cells were then lysed with ice-cold ddH_2_O and counted on plates. The experiment was performed in triplicate.

### Adhesion-related genes quanttatve real-time PCR (qPCR)

The experiments were performed using a previously described method [45]. The WT, Δ*pdh* and CΔ*pdh* strains 1:100 were transferred to fresh THB medium and cultured to log phase growth at 37°C. RNA from three strains was extracted using the TRIzon (CoWin Biosciences Co., Ltd, Biejing, china) according to the manufacturer’s instructions. RNA samples were reverse transcribed into cDNA using the PrimeScript RT-PCR kit (Takara). The amplification reaction of qPCR was carried out by the quantitative reagent SYBR Premix ExTaqTM. The internal reference gene is 16S rRNA (primer sequences are shown in Table 4). The reaction system was set to 20 μL, of which 2× Premix ExTaqTM II 10 μL, 0.5 μL each of the upstream and downstream primers, 1 μL of the cDNA template, and 6.0 μL of ddH_2_O. The PCR reaction conditions were set to 95°C for 10 min, 95°C for 15 s, 60°C for 1 min, 40 cycles, and three replicates for each gene. The relative expression level of the gene was determined by referring to the 2^−ΔΔCT^calculation method provided by Ma [46].

**Table 4.** Primers for qPCR used in this study.

Name	Sequence (5′-3′)	Target gene
16s RNA-S	GTTGCGAACGGGTGAGTAA	*16sRNA*
16s RNA-A	TCTCAGGTCGGCTATGTATCG	
CcpA-S	CGGTGTCAGTGATATGGG	*ccpA*
CcpA-A	GTCAGGTTTGGACGGGTA	
Fbps-S	AACCATCTTGCCAGGCTCCAC	*fbps*
Fbps-A	CAGTTCAGAAGCCGTATCCCGAC	
GapdH-S	CTTGGTAATCCCAGAATTGAACGG	*gapdH*
GapdH-A	TCATAGCAGCGTTTACTTCTTCAGC	
PerR-S	TTGAACACGTCATCCAACAT	*perR*
PerR-A	GTAGTTAGGTATTAGATCTTG	
Gor-S	GTTCACGCGCATCCTACG	*gor*
Gor-A	TACCAGGAATAGCAGGGAC	
Mrp-S	CAGGTAACATCAGAATCACCACTTTT	*mrp*
Mrp-A	AAGTTTTGTTTGAGCATCCTCTATAGC	
SrtA-S	GAACCGCAATCCCACCAAT	*srtA*
SrtA-A	AAAAGAATAAACAGGCTGAGACAACA	
Cps2-S	ATTGGTAGGCACTGTCGTTGGTC	*cps2*
Cps2-A	AGAACTTAGCATTGTTGCGGTGG	
Ef-S	TCCAATCACAGATCCAGATAGCG	*ef*
Ef-A	CTGACCCATTTGGACCATCTAAG	
Gdh-S	CACCTTTACCACCGCCGATTG	*gdh*
Gdh-A	GGAAATGTTCAAGTCAACCGTGG	

### Intracellular survival assays

To evaluate the anti-phagocytic ability of three strains. The assay was performed as the previous description with some minor modifications [31]. RAW264.7 cells at a concentration of 4 × 10^5^ cells/well were cultured in 24-well tissue culture plates until the monolayer was overgrown, and the culture medium was discarded. 10 MOI (1 × 10^7^ CFU/mL, 500 µL/well) bacterial suspension diluted with DMEM (containing 10% FBS) was added. Liquid infection for 1 h. Rinse 4 times with sterile PBS (avoid pipetting), add 1 mL of DMEM maintenance solution containing ampicillin (100 μg/mL) for 30 min to kill bacteria on the cell surface and discard the culture solution. The cells were washed twice with 100 μL of 0.25% trypsin, dried for 5–10 min, and 1 mL of ice water was added to the refrigerator at 4°C for 10 min to fully lyse the cells. The suspension after cell lysis was blown and transferred to a sterile 1.5 mL centrifuge tube. After a 10-fold serial dilution, the appropriate dilution was applied to the TSB agar plates. The number of viable bacteria associated with each sample was measured after 2 h, 4 h, 6 h and 8 h of incubation. The experiment was performed in triplicate.

### Statstcal analysis

Statistical analysis of in vitro and in vivo experiments was performed using GraphPad software. One-way analysis of variance (ANOVA) was used to analyze in vitro data and biofilm formation, and two-way ANOVA was performed on qRT-PCR results. For in vivo infection experiments, survival data were analyzed using the log rank test. Values of *P* < 0.05 were considered significant and all data were expressed as mean ± SD. ***P < 0.001; **P < 0.01; *P < 0.05.
